# Development of Cardiac Events and Functional Recovery Prediction Models for Pediatric Dilated Cardiomyopathy

**DOI:** 10.3389/fped.2021.736872

**Published:** 2021-08-27

**Authors:** Dong-Hee Kim, Eun Seok Choi, Bo Sang Kwon, Chun Soo Park, Seul Gi Cha, Jae Suk Baek, Jeong Jin Yu, Young-Hwue Kim, Tae-Jin Yun

**Affiliations:** ^1^Division of Pediatric Cardiac Surgery, Asan Medical Center, University of Ulsan College of Medicine, Seoul, South Korea; ^2^Division of Pediatric Cardiology, Asan Medical Center, University of Ulsan College of Medicine, Seoul, South Korea

**Keywords:** pediatric dilated cardiomyopathy, suvival, functional recovery, heart transplantation, cardiac event, myocarditis

## Abstract

**Background:** Since both the risk of death and the probability of spontaneous functional recovery (FR) coexist in association with pediatric dilated cardiomyopathy (DCMP), management should be based on individualized outcome predictions.

**Methods:** A single-center retrospective review of 105 pediatric patients (age at presentation ≤ 18 years) with DCMP, managed between 1994 and 2017, was performed. Logistic regression was conducted to identify variables associated with FR and cardiac events (CEs), i.e., death or heart transplantation (HTPL), within 2 years after initial presentation. Two outcome prediction models were formulated using these variables.

**Results:** Twenty-six (24.8%) and 51 patients (48.6%) experienced FR and CE, respectively, within 2 years after initial presentation. Predictors of mortality without HTPL were earlier era at presentation (HR: 4.13; 95% CI: 1.88–9.06; *p* < 0.001) and significant TR (≥moderate; HR: 4.31; 95% CI: 1.26–14.77; *p* = 0.020) in multivariable Cox regression model. Predictors of FR were recent era (HR: 4.49; 95% CI: 1.40–14.44; *p* = 0.0012), younger age at initial presentation (HR: 0.98 per 1 month increase; 95% CI: 0.97–0.99, *p* < 0.001), post-myocarditis DCMP (HR: 4.29; 95% CI: 1.32–13.93; *p* = 0.015), and arrhythmia-mediated DCMP (HR: 26.88; 95% CI: 2.61–276.70; *p* = 0.006). Risk factors for CEs was idiopathic DCMP (HR: 2.95; 95% CI: 1.32–6.56, *p* = 0.008). The low-risk group who had higher probability of FR than CE in prediction model had a slightly higher overall survival rate (71.4 vs. 52.2% at 10 years after presentation; log-rank *p* = 0.09) and a significantly higher HTPL-free survival rate (67.5 vs. 24.9% at 10 years after presentation; log-rank *p* < 0.001) than the high-risk group.

**Conclusions:** Prognostication and management strategies for pediatric DCMP may be enhanced by risk stratification using outcome prediction modeling.

## Graphical Abstract

Outcome Prediction Models for Pediatric Dilated Cardiomyopathy. The probabilities of FR and CEs within 2 years after the initial presentation can be calculated using outcome prediction models for pediatric patients with DCMP. Medical treatment with close follow-up is advisable for patients who are deemed more likely to experience FR than CEs, while early registration for HTPL and aggressive employment of LVADs is recommended for patients who are deemed more likely to experience CEs than FR. DCMP, dilated cardiomyopathy; FR, functional recovery of the left ventricle; CE, cardiac event; F/U, follow-up; LVAD, left ventricular assist device. 
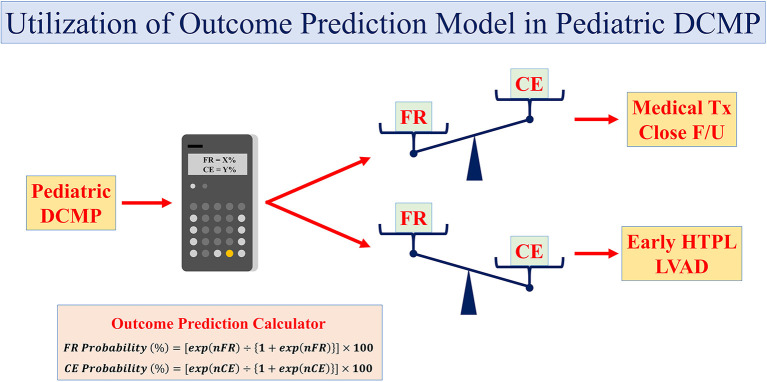

## Introduction

Dilated cardiomyopathy (DCMP), which is characterized by severe dysfunction and dilatation of the left ventricle, is the most common cardiomyopathic phenotype affecting children (1–4). The annual incidence of pediatric DCMP has been reported to be 0.57 cases per 100,000 per year in North America (5). Transplantation-free survival rates among pediatric DCMP patients remain poor despite recent advances in medical treatment and mechanical circulatory support (MCS) (6–9). Nearly half of pediatric DCMP patients die early or require early heart transplantation (HTPL) (5, 10, 11). HTPL outcomes have improved markedly among both adults and children in recent years, rendering HTPL a potent therapeutic option (7, 12, 13). However, children with DCMP may experience spontaneous functional recovery (FR) of the left ventricle, and HTPL tends to be reserved for critically ill patients during the early stage after initial diagnosis (14–16). Therefore, it is important to identify subsets of patients who are likely to have poor outcomes and those who are likely to recover spontaneously, given that both death and FR tend to occur relatively soon after the onset of symptoms. We sought to formulate outcome prediction models stratifying risk among pediatric DCMP patients to facilitate appropriate and individualized early management.

## Materials and Methods

### Patients

This single-center retrospective review analyzed data retrieved from the medical records of 105 pediatric patients ( ≤ 18 years of age at presentation) with DCMP who were managed at our institution between December 1994 and November 2017. Sixty patients (60/105, 57.1%) were male, and the median age at initial diagnosis was 2.19 years (interquartile range [IQR], 0.52 to 11.63). Medical record data were captured regarding demographic characteristics, familial history of sudden cardiac death or DCMP, medical treatments, and comorbidities.

### Diagnosis and Definitions

A diagnosis of DCMP was considered based on a patient's clinical history and physical examination and confirmed by pathognomonic echocardiographic findings of an abnormally dilated left ventricular cavity and left ventricular systolic dysfunction [i.e., a left ventricular ejection fraction (LVEF) <50%]. Although diagnoses were made mainly according to the phenotypic characteristics of DCMP (5, 10, 11, 17), potential underlying genetic abnormalities, such as Turner syndrome or Duchenne muscular dystrophy, were also investigated during data collection. Arrhythmia-mediated DCMP was defined as DCMP that developed after an attack of electrocardiographically documented tachyarrhythmias. Myocarditis-induced DCMP was defined as DCMP that developed after an episode of myocarditis, which was diagnosed by myocardial biopsy, cardiac magnetic resonance imaging, or laboratory findings (i.e., identification of viral markers or at least a 2-fold increase in serum cardiac enzymes at initial presentation). Patients without any demonstrable causes of DCMP were categorized as having idiopathic DCMP. FR was defined as recuperation from left ventricular dysfunction with an echocardiographically confirmed LVEF > 50%. The composite outcome of HTPL or death without HTPL was defined as cardiac events (CE). Study endpoints that occurred within 2 years of initial presentation were considered early FR or CEs.

### Treatment Strategy

In patients who were diagnosed as DCMP for the first time, echocardiography, electrocardiogram, and appropriate laboratory test including viral markers were performed to figure out the underlying etiology causing DCMP. Intravenous immunoglobulin (IVIG) treatment was indicated within the first week after the initial diagnosis for patients who were deemed to have myocarditis. Steroid pulse therapy was reserved for patients who did not respond to maximal medical therapy and IVIG. The use of inotropic agents, application of ECMO was determined by the patient's status, including vital signs and perfusion status. For the first time, we would like to expect functional recovery, especially in young patients. However, if there were no significant functional improvements despite several weeks of medical treatment, we would like to enlist the patients for HTPL. Upon the listing for HTPL, the patient's status, such as vital signs, and requirement of inotropic or ECMO, was also considered. For example, patients with profound deterioration would be registered for HTPL promptly. Outpatient-based medications, including digoxin, angiotensin-converting enzyme inhibitors, beta-blockade, antiarrhythmic agents, and anticoagulants, were not accounted for in the statistical analysis because the prescription timing, duration of administration, and drug combinations varied widely among the patients.

### Statistical Analysis

All categorical variables are expressed as frequencies with percentages, and continuous variables are expressed as means with standard deviations or medians with IQRs. Kaplan–Meier analysis was used to estimate survival or freedom from time-related events, and differences between the groups were compared using the log-rank test. A Cox proportional hazard model was fitted to identify the risk factors for decreased time to death without HTPL, considering the performance of HTPL as a censored event. Variables with a *p*-value of < 0.1 on a univariable Cox regression were included in the multivariable analysis. To develop predictive models for FR or CEs during a specific time frame after initial presentation, we identified predictors of FR and risk factors for CE within 2 years after initial presentation using logistic regression analysis, including variables with a univariable *p*-value of < 0.1 in the multivariable analysis. Consequently, the probability of FR or CEs within 2 years after initial presentation was estimated using the statistically significant predictors of FR or CEs identified from the logistic regression analysis.

The accuracy of the calculated probability was validated with the receiver operating characteristics curve. Differences in the final analysis were regarded as statistically significant if *p*-values were < 0.05. R software version 3.6.1 (www.r-project.org) was used for analysis.

## Results

### Patient Characteristics

Thirty-five patients (35/105, 33.3%) had DCMP induced by myocarditis, seven patients (7/105, 6.6%) had arrhythmia-mediated DCMP, and five patients (5/105, 4.8%) had doxorubicin-induced DCMP, and the remaining 58 patients (58/105, 55.2%) were categorized as having idiopathic DCMP. Three patients had underlying genetic disorders (namely, Turner syndrome, Seckel syndrome, and mitochondrial disease), and one patient had Duchenne muscular dystrophy. Six patients (6/105, 5.7%) had a familial history of sudden death or DCMP. The median LVEF at diagnosis was 25.5% (IQR: 17.5–31.0%). Fifteen patients (15/105, 14.3%) required extracorporeal membrane oxygenation (ECMO) at a median of 12 days (IQR: 0–50 days) after initial presentation, among whom nine patients (9/15, 60%) underwent HTPL (seven patients on ECMO, 2 patients off ECMO). An additional 19 patients underwent HTPL without a history of ECMO support. Thus, HTPL was performed for 28 patients (28/105, 26.7%) at a median of 6 months (IQR: 1.9–27 months) after initial presentation.

### Clinical Outcomes and Predictors of FR and CE Occurrence

**Figure 1:**
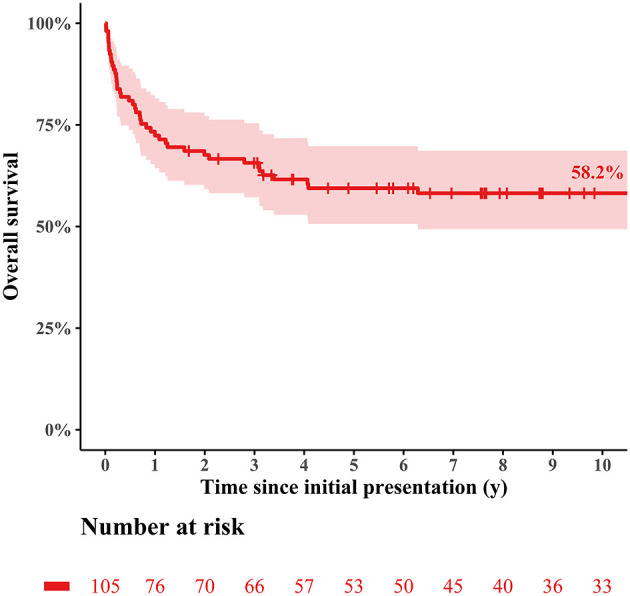
Overall survival of the entire cohort.

**Figure 2:**
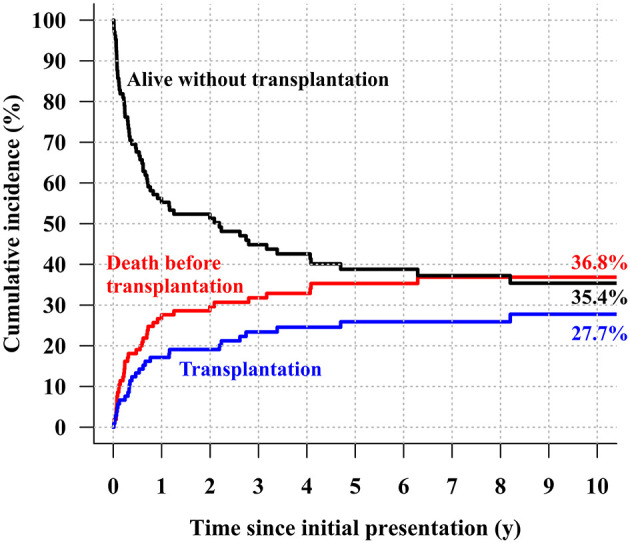
Cumulative incidence of transplantation-free survival, death before heart transplantation, and heart transplantation.

**Figure 3:**
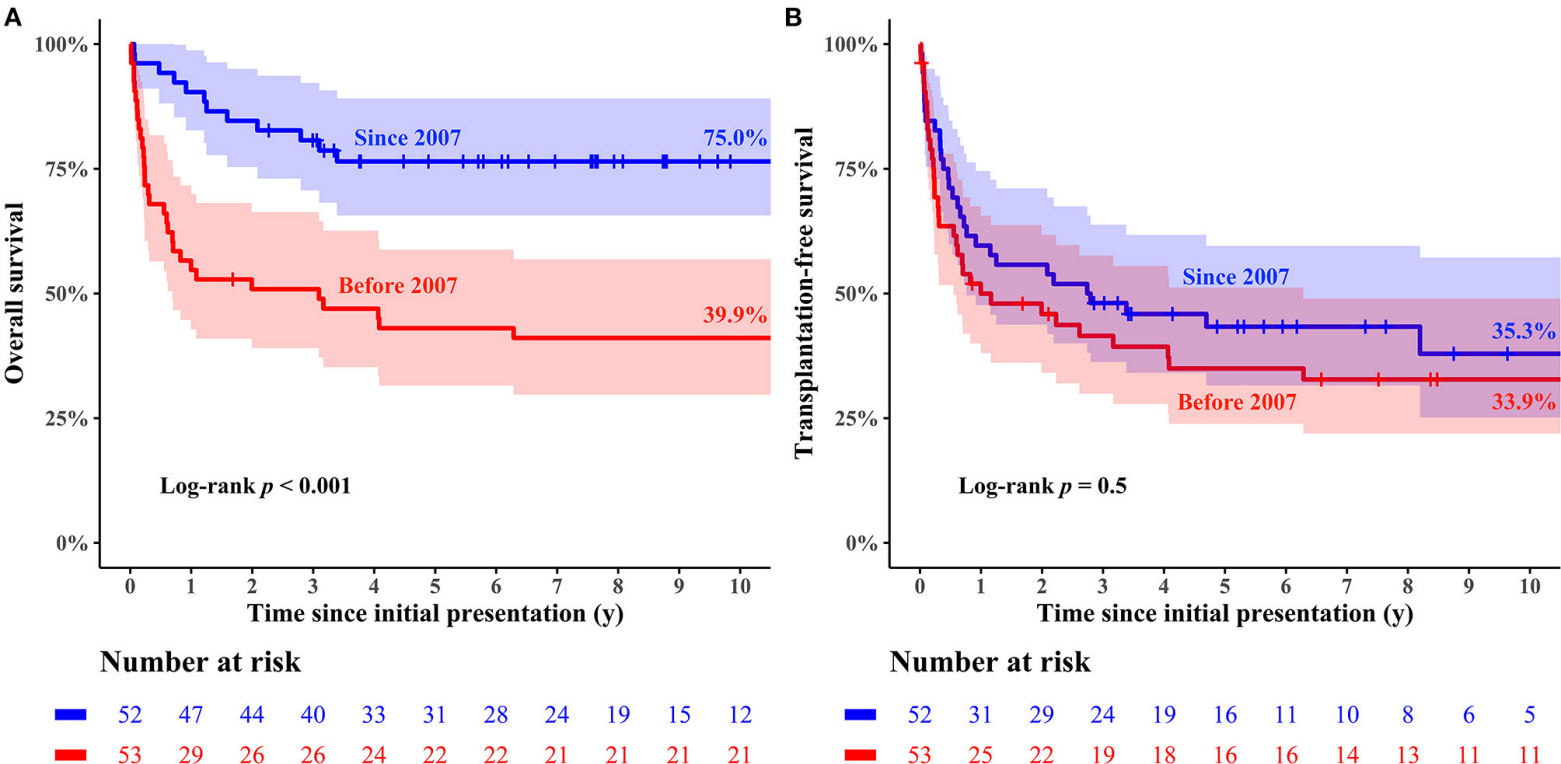
Kaplan–Meier curves of clinical outcomes stratified by eras at initial presentation. **(A)** overall survival, **(B)** transplantation-free Survival.

The median follow-up duration was 5.5 years (range: 0.1–25.1 years), during which 49 deaths (49/105, 46.7%; 39 deaths without HTPL and 10 deaths after HTPL) occurred. The overall survival rate at 10 years after initial presentation was 58.2% (Figure 1). The cumulative incidence curves in Figure 2 show that 36.8% of the patients died without HTPL, 27.7% underwent HTPL, and the remaining 35.4% were alive without HTPL 10 years after initial presentation. Figure 3A illustrates overall survival according to the eras of presentation, showing that there was a significant survival improvement from 2007 onward (75.0 vs. 39.9% at 10 years; log-rank *p* < 0.001). However, as Figure 3B illustrates, there was no significant difference in HTPL-free survival between the 2 time frames (35.3 vs. 33.9%; log-rank *p* = 0.5). Inter-era differences in patient characteristics are summarized in Table 1. The univariable Cox regression analysis yielded the following risk factors for decreased time to death without HTPL: earlier era at presentation (presentation before 2007, *p* < 0.001) and significant tricuspid regurgitation (TR) at presentation (TR ≥ moderate; *p* = 0.033). After multivariable analysis, earlier era at presentation (HR: 4.13; 95% CI: 1.88–9.06; *p* < 0.001) and significant TR (≥moderate; HR: 4.31; 95% CI: 1.26–14.77; *p* = 0.020) remained significant (Table 2).

**Table 1:** Comparison of the patient characteristics between the two eras.

	**Overall** **(*N =* 105)**	**Before 2007** **(*N =* 52)**	**Since 2007** **(*N =* 53)**	***p***
Sex (male)	60 (57.1)	32 (61.5)	28 (52.8)	0.48
Age at presentation (year, median [IQR])	2.19 (0.52–11.62)	6.12 (0.37–12.54)	1.97 (0.53–10.54)	0.61
Familial history	6 (5.7)	6 (11.5)	0 (0.0)	0.033
Genetic disease	3 (2.9)	2 (3.8)	1 (1.9)	0.99
Neuromuscular disease	1 (1.0)	1 (1.9)	0 (0.0)	0.99
Post-myocarditis	35 (33.3)	16 (30.8)	19 (35.8)	0.73
Adriamycin-induced	5 (4.8)	4 (7.7)	1 (1.9)	0.35
Arrhythmia-mediated	7 (6.7)	4 (7.7)	3 (5.7)	0.98
Idiopathic	58 (55.2)	28 (53.8)	30 (56.6)	0.93
LVEF (%, median [IQR])	25.96 (17.50–31.00)	23.90 (16.58–30.03)	27.00 (19.50–32.00)	0.065
MR ≥ moderate	15 (14.3)	8 (15.4)	7 (13.2)	0.97
TR ≥ moderate	5 (4.8)	2 (3.8)	3 (5.7)	>0.99
IVIG	33 (31.4)	21 (40.4)	12 (22.6)	0.080
Steroid pulse therapy	6 (5.7)	5 (9.6)	1 (1.9)	0.20
CRT	3 (2.9)	3 (5.8)	0 (0.0)	0.24
ECMO	15 (14.3)	15 (28.8)	0 (0.0)	<0.001
HTPL	28 (26.7)	22 (42.3)	6 (11.3)	0.001
Functional recovery within 2 years	26 (24.8)	17 (32.7)	9 (17.0)	0.101

*IQR, interquartile range; LVEF, left ventricular ejection fraction; MR, mitral regurgitation; TR, tricuspid regurgitation; IVIG, intravenous immunoglobulin; CRT, cardiac resynchronization therapy; ECMO, extracorporeal membrane oxygenator; HTPL, heart transplantation*.

**Table 2:** Risk factors for decreased time to death before heart transplantation.

	**Univariable**			**Multivariable**		
**Variables**	**HR**	**95% CI**	***p***	**HR**	**95% CI**	***p***
Sex (male)	1.29	0.69–2.43	0.43			
Earlier era (presentation before 2007)	4.00	1.83–8.76	<0.001	4.13	1.88–9.06	<0.001
Age at initial presentation (months)	1.00	1.00–1.01	0.49			
Familial history	1.32	0.31–5.51	0.71			
Genetic disease	1.82	0.44–7.58	0.41			
Neuromuscular disease	2.39	0.33–17.50	0.39			
Post-myocarditis	1.17	0.61–2.23	0.64			
Adriamycin-induced	NA		>0.99			
Arrhythmia-mediated	NA		>0.99			
Idiopathic	1.66	0.87–3.18	0.13			
LVEF (%, median [IQR])	1.00	0.97–1.04	0.89			
MR ≥ moderate	1.09	0.46–2.61	0.84			
TR ≥ moderate	3.69	1.11–12.24	0.033	4.31	1.26–14.77	0.020
IVIG	0.74	0.36–1.52	0.42			
Steroid pulse therapy	0.91	0.22–3.77	0.89			
CRT	0.71	0.10–5.20	0.74			
ECMO	0.97	0.34–2.74	0.95			

*HR, hazard ratio; CI, confidence interval; LVEF, left ventricular ejection fraction; MR, mitral regurgitation; TR, tricuspid regurgitation; IVIG, intravenous immunoglobulin; CRT, cardiac resynchronization therapy; ECMO, extracorporeal membrane oxygenation*.

**Figure 4:**
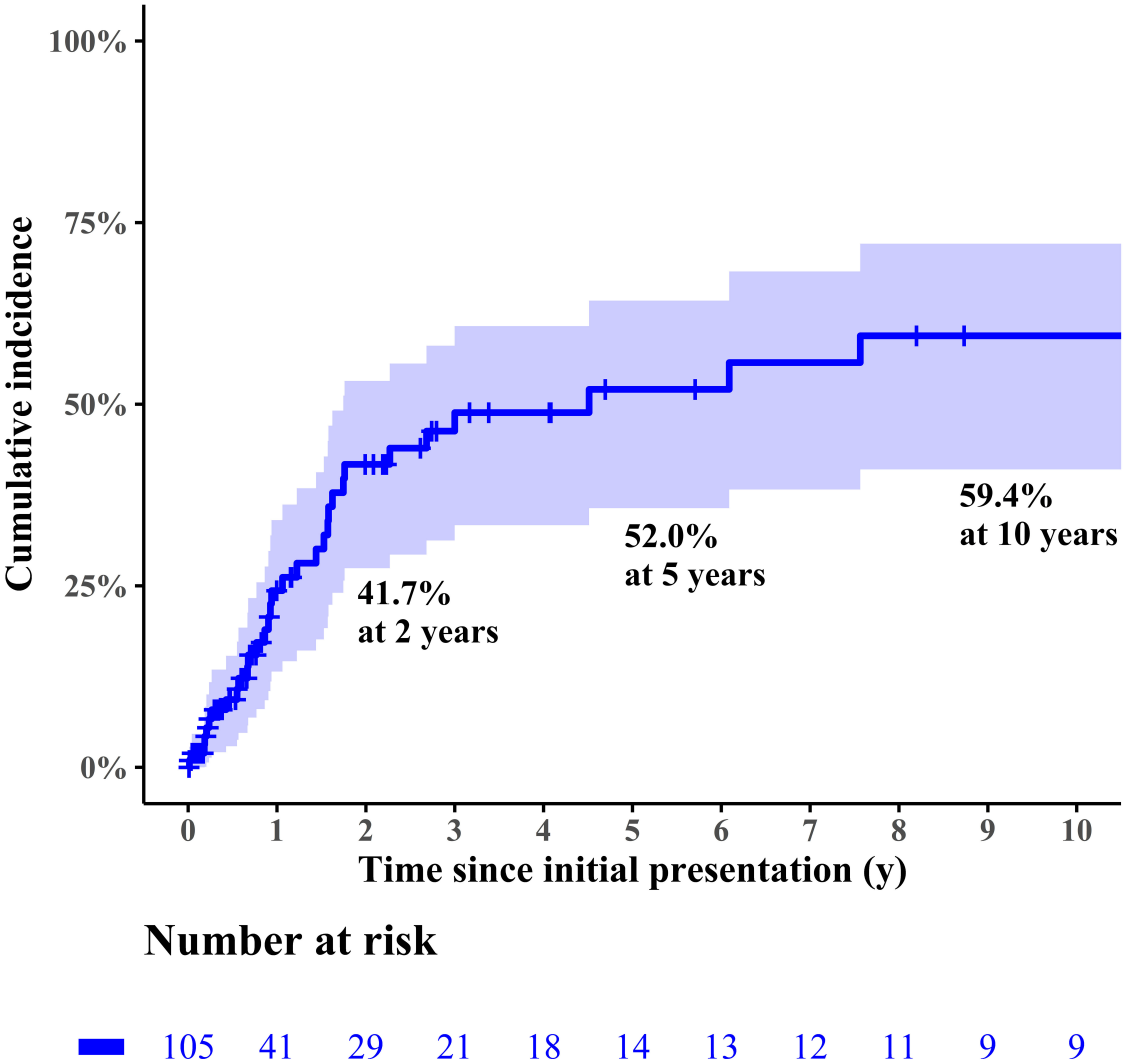
Cumulative incidence of functional recovery in the entire cohort.

**Figure 5:**
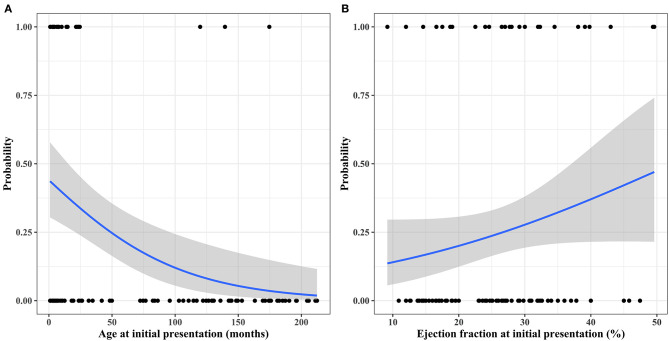
Probability curves for functional recovery within 2 years after initial presentation according to **(A)** age at initial presentation and **(B)** initial left ventricular ejection fraction.

During follow-up, 33 patients (33/105, 31.4%) experienced FR at a median interval of 11.4 months (IQR: 5.9–21.3 months) after initial presentation. The cumulative incidence of FR is depicted in Figure 4. Two patients (2/33, 6.1%) experienced deterioration of left ventricular function after FR; these patients were 2.7 and 7.6 months old at presentation and exhibited mild deterioration of left ventricular function (LVEF of 48 and 42%) at 79 and 67 months after FR, respectively. Two patients died of noncardiac causes after FR. An 18-month-old girl, who was 3 months old at presentation and experienced FR 8 months after her initial presentation, died of acute respiratory distress syndrome 7 months after FR. A 6.8-year-old girl, who was 6 months old at presentation and experienced FR 4.5 years after her initial presentation, died of an acute exacerbation of lobar pneumonia 21 months after FR. FR took place within 2 years after the initial presentation in 26 patients (26/105, 24.8%). Univariable logistic regression analysis found recent era (presentation since 2007; *p* = 0.066), younger age at initial presentation (*p* < 0.001), post-myocarditis DCMP (*p* = 0.041), arrhythmia mediated DCMP (*p* = 0.011), higher LVEF at initial presentation (*p* = 0.083), non-idiopathic DCMP (*p* = 0.005), and use of IVIG (*p* = 0.006) to be predictors for FR within 2 years after initial presentation, albeit some of these factors were not statistically significant. The estimated probability curves for FR according to the age at presentation and initial LVEF are illustrated in Figure 5. Multivariable analysis revealed the following independent predictors of FR within 2 years after initial presentation: recent era (HR: 4.49; 95% CI: 1.40 to 14.44; *p* = 0.012), younger age at initial presentation (HR: 0.98 per 1 month increase; 95% CI: 0.97–0.99, *p* < 0.001), post-myocarditis DCMP (HR: 4.29; 95% CI: 1.32–13.93; *p* = 0.015), and arrhythmia-mediated DCMP (HR: 26.88; 95% CI: 2.61–276.70; *p* = 0.006) (Table 3).

**Table 3:** Predictors of functional recovery within 2 years after initial presentation.

	**Univariable**			**Multivariable**		
**Variables**	**HR**	**95% CI**	***p***	**HR**	**95% CI**	***p***
Sex	0.63	0.25–1.59	0.33			
Earlier era (presentation before 2007)	0.42	0.17–1.06	0.066	0.22	0.07–0.72	0.012
Age at initial presentation (month)	0.98	0.97–0.99	<0.001	0.98	0.97–0.99	<0.001
Familial history	NA		0.99			
Genetic disease	NA		0.99			
Neuromuscular disease	NA		0.99			
Post-myocarditis	2.59	1.04–6.45	0.041	4.29	1.32–13.93	0.015
Adriamycin-induced	NA		0.99			
Arrhythmia-mediated	9.17	1.66–50.65	0.011	26.88	2.61–276.70	0.006
Idiopathic	0.26	0.10–0.67	0.005	NA		
LVEF	1.04	0.99–1.10	0.083	NA		
MR ≥ moderate	1.12	0.32–3.89	0.85			
TR ≥ moderate	NA		0.99			
IVIG	3.68	1.46–9.32	0.006	NA		
Steroid pulse therapy	0.59	0.07–5.31	0.64			
CRT	NA		0.99			
ECMO	0.73	0.19–2.81	0.65			

*HR, hazard ratio; CI, confidence interval; LVEF, left ventricular ejection fraction; MR, mitral regurgitation; TR, tricuspid regurgitation; IVIG, intravenous immunoglobulin; CRT, cardiac resynchronization therapy; ECMO, extracorporeal membrane oxygenation*.

**Figure 6:**
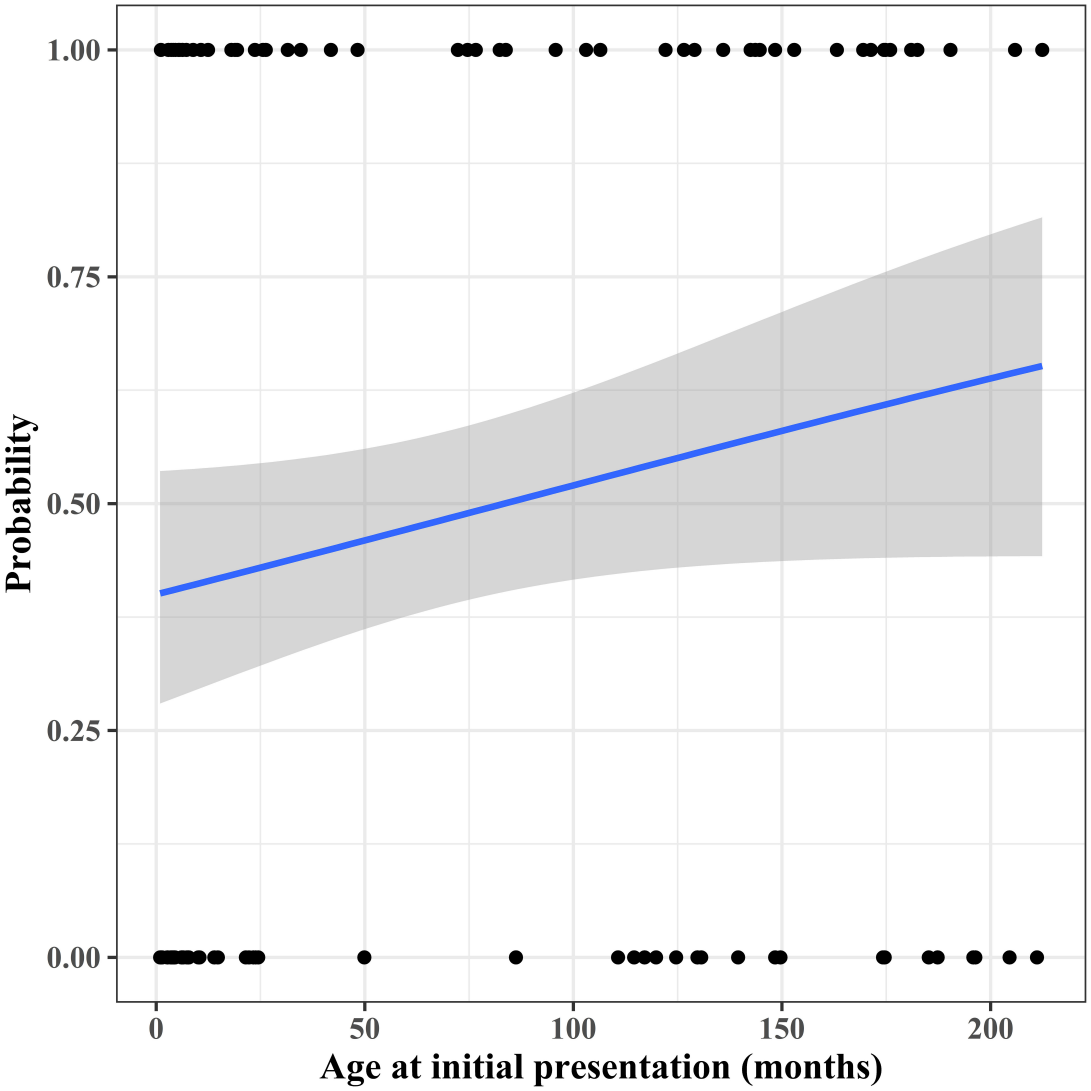
Probability curve for cardiac events within 2 years after initial presentation according to age at initial presentation.

Within 2 years after initial presentation, 51 patients (51/105, 48.6%) had CEs: 31 patients (31/105, 29.5%) died without HTPL and 20 patients (19.0%) underwent HTPL. According to the univariable analysis, predictors of CEs within 2 years after presentation were older age at initial presentation (*p* = 0.080), idiopathic DCMP (*p* = 0.008), and the need for ECMO (*p* = 0.047). Estimated probability curves for CEs according to age at initial presentation are illustrated in Figure 6. Only idiopathic DCMP was an independent risk factor for CEs in multivariable analysis (Table 4).

**Table 4:** Risk factors for cardiac events within 2 years after initial presentation.

	**Univariable**			**Multivariable**		
**Variables**	**HR**	**95% CI**	***p***	**HR**	**95% CI**	***p***
Sex	0.88	0.40–1.90	0.74			
Earlier era (presentation before 2007)	1.41	0.65–3.04	0.38			
Age at initial presentation (month)	1.005	1.00–1.01	0.080	NA		
Familial history	2.21	0.39–12.64	0.37			
Genetic disease	0.52	0.05–5.92	0.60			
Neuromuscular disease	NA		0.99			
Post-myocarditis	0.71	0.31–1.60	0.41			
Adriamycin-induced	0.25	0.03–2.32	0.22			
Arrhythmia mediated	NA		0.99			
Idiopathic	2.95	1.32–6.56	0.008	2.95	1.32–6.56	0.008
LVEF	0.99	0.95–1.03	0.54			
MR ≥ moderate	0.91	0.31–2.74	0.87			
TR ≥ moderate	4.51	0.49–41.79	0.19			
IVIG	0.99	0.44–2.27	0.99			
Steroid pulse therapy	1.06	0.20–5.52	0.94			
CRT	0.52	0.05–5.92	0.60			
ECMO	3.44	1.02–11.62	0.047	NA		

*HR, hazard ratio; CI, confidence interval; LVEF, left ventricular ejection fraction; MR, mitral regurgitation; TR, tricuspid regurgitation; IVIG, intravenous immunoglobulin; CRT, cardiac resynchronization therapy; ECMO, extracorporeal membrane oxygenation*.

### Formulation of the Outcome Prediction Model

Outcome prediction models were formulated from the predictors of FR or CEs within 2 years after the initial presentation identified by the multivariable logistic regression analyses (Tables 3, 4). As the date of the initial presentation could not be used for the prospective probability model for FR, the era effect was not used to formulate the equations. Therefore, age, post-myocarditis DCMP, and arrhythmia-mediated DCMP were used for the FR probability equation. Similarly, idiopathic DCMP was used for the CE probability equation. Continuous variables, such as age in months, were entered into the equations as numbers, and the presence or absence of the categorical variables was treated as 1 or 0, respectively. Equations for calculating the probability of each event are available in the Appendix.

**Figure 7:**
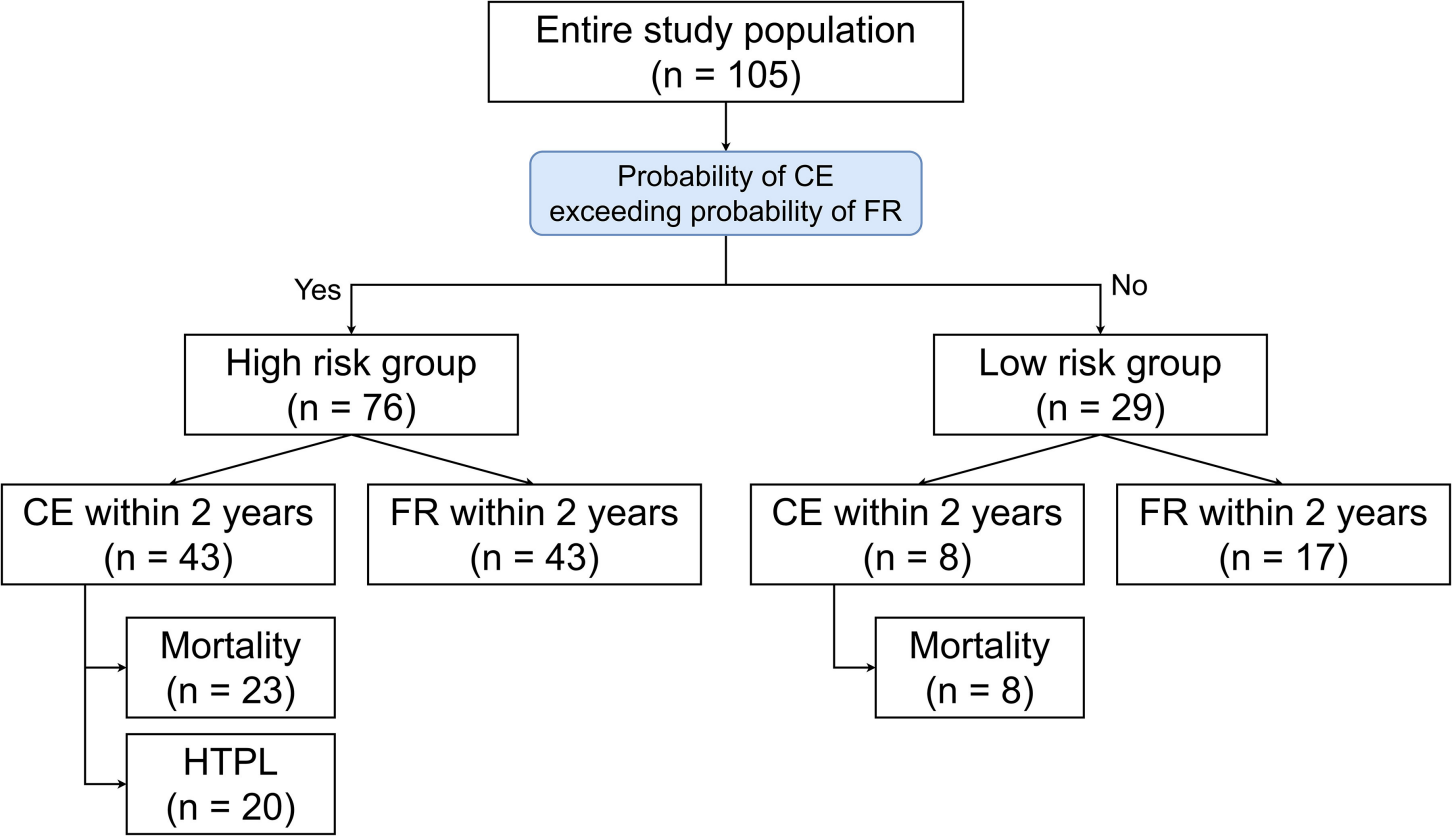
Observed events after stratification into risk groups according to the calculated probability. CE, cardiac event; FR, functional recovery.

**Figure 8:**
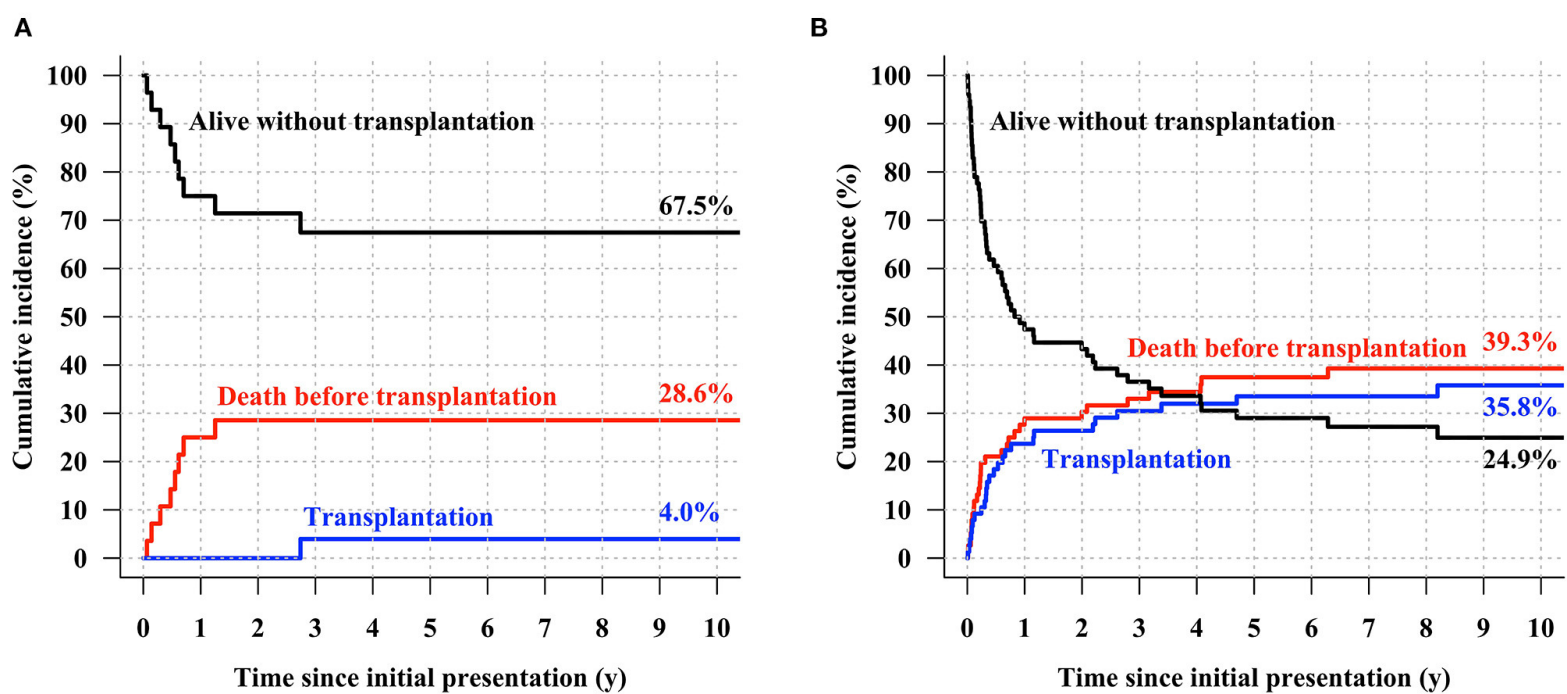
Cumulative incidence of transplantation-free survival, death before heart transplantation, and heart transplantation in **(A)** the low-risk group and **(B)** high-risk group.

**Figure 9:**
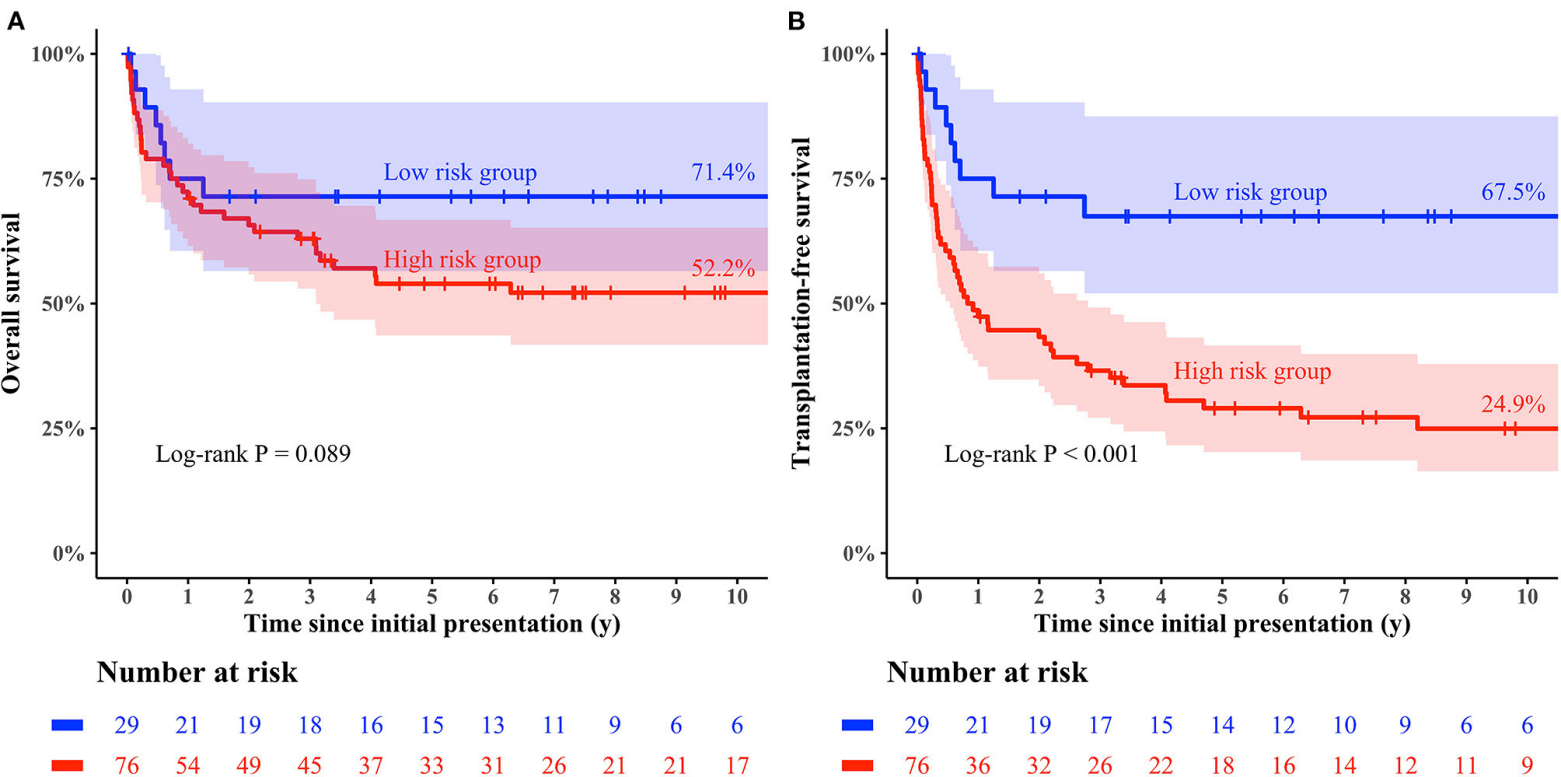
Kaplan–Meier curves for clinical outcomes stratified by risk groups **(A)** overall survival, **(B)** transplantation-free survival.

Using the area under the receiver operating characteristics curve, the calculated probability of FR and CE showed prediction accuracy of 0.830 (95% CI, 0.740–0.920) and 0.630 (95% CI: 0.538–0.723), respectively. Risk-stratification was attempted for the entire cohort using the 2 outcome prediction models, and patients were categorized into two groups: the low-risk group (*N* = 29, 27.6%) was defined as the subset whose FR probabilities were higher than their CE probabilities, while the high-risk group (*N* = 76, 72.4%) comprised the subset whose CE probabilities were higher than their FR probabilities. In the high-risk group, CE was observed in 43 patients (43/72, 59.7%), while eight patients (8/29, 27.6%) had CE in the low-risk group within 2 years (Figure 7). The cumulative incidence curves for the low-risk group showed that 4.0% of the low-risk patients underwent HTPL, 28.6% died without HTPL, and 67.5% were alive without HTPL at 10 years after initial presentation (Figure 8A). The curves for the high-risk group showed that 35.8% of these patients underwent HTPL, 39.3% died without HTPL, and 24.9% were alive without HTPL at 10 years after initial presentation (Figure 8B). The low-risk group had a slightly higher overall survival rate (71.4 vs. 52.2% at 10 years after initial presentation; log-rank *p* = 0.09; Figure 9A) and a higher HTPL-free survival rate (67.5 vs. 24.9% at 10 years after initial presentation; log-rank *p* < 0.001; Figure 9B) than the high-risk group.

## Discussion

This study showed that the prognosis of pediatric patients with DCMP is still suboptimal in that a significant number of patients die early while waiting for HTPL. Although overall survival rates have markedly improved (18), transplantation-free survival rates have not changed much, which signifies that improvements in overall survival are mainly attributable to appropriately indicated and timely HTPL (13, 19). In our study, there was a significant survival improvement from 2007; however, there was no significant difference in HTPL-free survival between the 2-time frames, signifying that improvement in overall survival in the latter era was mainly attributed to the more aggressive application of HTPL. Notably, many patients with initially compromised left ventricular function experience FR of the left ventricle. Because both CEs and FR occur most frequently within 2 years after initial presentation, prognostication and management strategies should be based on proper risk stratification during this early stage. To this end, we sought to develop outcome prediction models for both CEs and FR. If the probability of CEs is higher than that for FR for a patient with DCMP, early registration for preemptive HTPL (or ventricular assist device) may be suitable. In the reverse scenario, the patient would benefit from continuing medical treatment with anticipation of FR.

Given the donor shortage and the high frequency of rapid clinical deterioration after the initial presentation in the pediatric population, registration for HTPL should be done far in advance once a patient is deemed to have a high CE probability (14–16, 20). Various risk factors for death among pediatric patients with DCMP have been highlighted in the previous studies, such as older age, profound cardiac dysfunction, severe ventricular dilatation, and the need for hospitalization at initial presentation (3, 5, 10, 14, 20–24). This study additionally found that idiopathic DCMP is a significant risk factor for CEs within 2 years after presentation. However, Cox regression analysis showed moderate to severe TR at initial presentation was the only significant risk factor for decreased time to death without HTPL. Significant TR could be a result of pulmonary hypertension caused either by severe left ventricular dysfunction (15) or by right ventricular dysfunction *per se* in patients with advanced DCMP (25).

We identified certain causes of DCMP (i.e., myocarditis and arrhythmia) as predictors of FR, as similarly indicated in previous studies (3, 11, 21, 26, 27). Appropriate and timely use of cardiac resynchronization therapy, which has been indicated for adults with severe heart failure (28), was not identified as a predictor of FR in this study. However, younger age at initial presentation was identified as a predictor of FR in this study, as indicated in other studies (23, 29). In the subgroup of patients aged <2 years at presentation (*n* = 50), a significant number of patients experienced FR (*N* = 27, 54%), especially within 2 years after initial presentation (*N* = 22, 44%). Therefore, the probability of FR compared with the probability of CE occurrence within 2 years should be carefully assessed before registering younger patients for HTPL. Given that deterioration of left ventricular function may occur after FR, vigilant outpatient monitoring and frequent reassessment of cardiac function are vital even for patients with FR.

There were several limitations to this study. Concerning the causes of DCMP, the high incidence of idiopathic DCMP in this study may be attributable, at least in part, to the retrospective study design and consequent missing clinical information. Without pathognomonic findings of myocarditis, tachyarrhythmia, or a clinical history of the use of cardiotoxic drugs, the cause of DCMP was deemed idiopathic. Moreover, improvements in laboratory techniques used to diagnose myocarditis, such as the assessment of troponin I level instead of lactate dehydrogenase or creatinine kinase levels, further complicated the task of identifying underlying causes. Although it is indisputable that HTPL is a final therapeutic option for end-stage DCMP, it is unclear whether HTPL was performed in a timely fashion for appropriately selected patients. We included the HTPL into CE because these patients might not be survived in the clinical situation. However, the decision of whether to have the HTPL could be influenced by other factors.

Furthermore, the impact of medical treatment on the deferment of HTPL or improvements in clinical condition before HTPL could not be evaluated due to the multiple drug combinations and frequent regimen changes during the study period. Lastly, some may argue that this study cohort may not be representative because there were no patients treated with left ventricular assist devices (LVADs), which have been reported to be either an optimal supportive measure for bridging to HTPL (7, 12) or an ideal definitive treatment modality (i.e., a potent alternative to HTPL) for facilitating FR among DCMP patients (6, 9, 12). LVAD might have modified the clinical course for many study patients who died without HTPL by rescuing patients from impending cardiac death, stabilizing the pre-HTPL state among patients with multi-organ dysfunction (8), and avoiding ECMO-related complications (7, 12). However, given that application of LVADs would have been indicated for critically ill patients who were more likely to experience CEs than FR, the predictability of CEs in a cohort with LVADs may have been similar to that of a cohort without LAVDs if the application of LVADs or other forms of MCS was included in the CE definition.

## Data Availability Statement

The raw data supporting the conclusions of this article will be made available by the authors, without undue reservation.

## Ethics Statement

The studies involving human participants were reviewed and approved by Asan Medical Center Institutional Review Board. Written informed consent from the participants' legal guardian/next of kin was not required to participate in this study in accordance with the national legislation and the institutional requirements.

## Author Contributions

T-JY and CP contributed to the conception and design. SC, JB, JY, and Y-HK were involved in the data collection and provision of resources. EC, BK, and D-HK analyzed and interpreted the results. D-HK wrote a preliminary version of the article and T-JY revised it. All co-authors reviewed and approved the manuscript.

## Conflict of Interest

The authors declare that the research was conducted in the absence of any commercial or financial relationships that could be construed as a potential conflict of interest.

## Publisher's Note

All claims expressed in this article are solely those of the authors and do not necessarily represent those of their affiliated organizations, or those of the publisher, the editors and the reviewers. Any product that may be evaluated in this article, or claim that may be made by its manufacturer, is not guaranteed or endorsed by the publisher.

## Appendix

### Logistic Regression Equations for Calculating Event Probability

$$\begin{array}{l} \text{ Probability }\left(\%\right)\text{=}\left[\exp\left(\text{n}\right)÷\left\{\text{1+}\exp\left(\text{n}\right)\right\}\right]\times \text{100}\\ \;\left(n\right)=a+b1\times x1+\ldots +bk\times xk\\ \;\exp\left(n\right)=e^{a}\times {\left(e^{b1}\right)}^{x1}\times \ldots \times {\left(e^{bk}\right)}^{xk}\\ Probability\;of\;FR\\ within\;2\;years\;\left(\%\right)=\left[\exp\left(nFR\right)÷\left\{1+\exp\left(nFR\right)\right\}\right]\times 100\\ \;nFR=\left(-1.000\right)+\left(-0.018\times age\;in\;months\right)\\ \;+\left(1.213\times postmyocarditis\;DCMP\right)\\ \;+\left(3.106\times arrhythmia\;mediated\;DCMP\right)\\ \;\exp\left(nFR\right)=0.368\times 0.983^{age\;\left(months\right)}\\ \;\times 3.362^{postmyocarditis\;DCMP}\\ \;\times 22.333^{arrhythmia\;mediated\;DCMP}\\ \;Probability\;of\;\\ \;CE\;occurrence\\ within\;2\;years\left(\%\right)=\left[\exp\left(nCE\right)÷\left\{1+\exp\left(nCE\right)\right\}\right]\times 100\\ \;nCE=\left(-0.661\right)+\left(1.081\times idiopathic\;DCMP\right)\\ \;\exp\left(nCE\right)=0.516\times 2.948^{idiopathic\;DCMP} \end{array}$$
